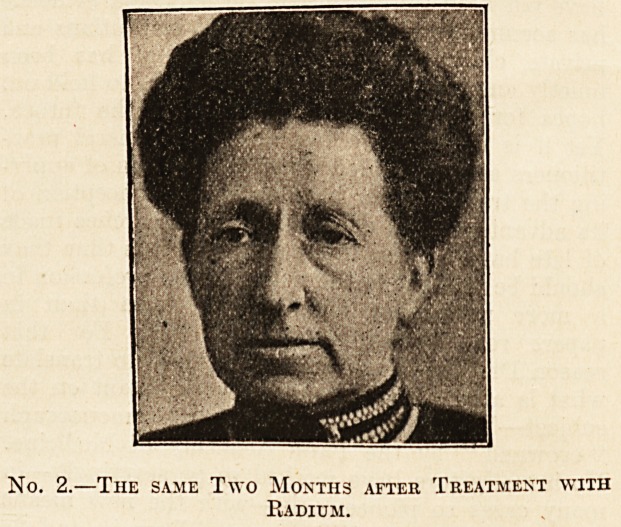# A New Field of Medical Treatment
**Radium Therapy,* by Dr. Louis Wickham and Dr. Degrais. Translated by Dr. Ernest Dore. With a preface by Sir Malcolm Morris. Cassell and Co., Ltd., London, New York, Toronto, and Melbourne. 1910. 15s. net.


**Published:** 1910-10-01

**Authors:** 


### SPECIAL ARTICLES.

### A NEW FIELD OF MEDICAL TREATMENT.*

### WHAT RADIUM HAS ACHIEVED AND WHAT IT MAY DO.

To the practitioner the possibilities of radium treatment are not entirely novel. During the last few years the element has been so extensively "boomed" that it was only natural for many to have anticipated the most brilliant results from its use in medicine and surgery. Doubtless these anticipations were too high, for at any rate they have not been fully realised; yet sufficient evidence has accumulated, in the records of institutions and private clinics where the new metal has been quietly and systematically worked with, to hold out hopes for more heartening results in the future. Yet it is even now a fact that few general practitioners are acquainted with the methods of applying the treatment, or have a definite conception of its advantages and dangers. The researches made of late have been so interesting that it is time they should be brought to the notice of the profession in a more permanent and suitable form than in papers read before learned societies. For that reason Dr. Dore has been well advised to translate what is after all the standard work extant on the subject—Wickham and Degrais' monograph "crowned" by the Paris Academy of Medicine. No investigators have worked so perseveringly—in many cases so pioneeringly—with the new means of treatment as Drs. Wickham and Degrais, and we owe much to them for the light they have thrown upon many obscure points, and chiefly for the frank and fair manner in which they have put forth their results.

### What Radium Is.

What is radium? It is possible that many of us will not be able to answer this question, not as the scientist expects an answer (for the element is still in some respects a scientific anomaly, as difficult to define in a few words as the natural order Orchidaceæ) but in simple commonplace language. Briefly put, as Dr. Wickham puts it, radium is a metal emitting radiations, which may be compared to a flow of electrified corpuscles, with a velocity equalling that of light. These corpuscles are so light that a speck of the metal may emit them for thousands of millions of years without appreciably diminishing in weight. When they come into contact with an electroscope they discharge it; when they strike certain bodies they illuminate them. The metal gives out both light and heat, and the source of this heat is still an unsolved problem. Radium is an element, an alkaline earth metal, akin to barium and strontium, with a characteristic spectrum and an atomic weight of 226.45, and the history of its discovery is a page in the romance of chemistry. For a full narration of it we must refer our readers to Dr. Wickham's interesting chapter on radio-active substances.

### How it is Used.

In medicine, radium is used in the form of one of its salts, chloride, bromide, sulphate, carbonate, and so on. Most of the radium used in France comes from the Armet de Lisle works, where the various radio-active ores, of which varieties of pitchblende and autunite are the chief, are ground mechanically, and then prepared in an elaborate way so as to obtain a solution which is radio-active. This solution is further concentrated by a process known as fractionisation, the radium being crystallised out as a chloride or bromide. The whole process is exceedingly intricate. To obtain two centigrammes

* *Radium Therapy*, by Dr. Louis Wickham and Dr. Degrais. Translated by Dr. Ernest Dore. With a preface by Sir Malcolm Morris. Cassell and Co., Ltd., London, New York, Toronto, and Melbourne. 1910. 15s. net.

of radium chloride no less than a ton of ore and 50 tons of water are needed. The final residue possesses the following properties: it emits light and heat, it has the power of colouring certain substances such as glass, diamonds, etc., when allowed to act upon them; it affects a sensitive photographic plate; it renders air a good conductor of electricity; it produces phosphorescence in certain substances and emits rays that traverse opaque bodies; it destroys the germinating power of seeds, modifies growth in organisms; has powerful bactericidal properties; and produces analgesia without surface reaction. Its

No. 1.—Ulcerated Epithelioma of Forehead before Treatment.

powerful action on human tissues was accidentally discovered by M. Becquerel, who incautiously carried a tube containing pure radium in his waistcoat pocket. Henceforth the "Becquerel burn" will rank with Newton's apple and the historic parsley leaf!

Messrs. Wickham and Degrais have used both radium emanations and radiation. For a description of the apparatus we must again refer to the book. It may be mentioned, however, that they have found a convenient method of using their powerful remedy in the form of radiferous water and oil. The appliances generally used in London consist of metal discs or bases, usually of roughened copper, covered with radio-active varnish. This is the applicator, and is made in various models to facilitate exposure of level and concave or convex surfaces. Special instruments have been devised for uterine cases and for applying the emanations to deeply seated organs. The covering varnish is first melted and then a small quantity (usually estimated at one centigramme to the square centimetre of surface) is incorporated with it. The technique of application is too complicated to be here described; those interested in the various methods employed will find exhaustive instructions in the book itself.

### What Radium has Done.

Coming to the clinical results we find that the investigations have been productive of a vast mass of interesting material which is fully explained and elucidated by the authors. The illustrative cases, with photographs, that are a feature of the book, give a clear account of the clinical progress of certain lesions when submitted to the action of the metal. The chief successes have so far been obtained with carcinomata, keloids and scars, angiomata, pigmentary nævi, muco-cutaneous tuberculosis, skin affections, and in gynæcological cases. On page 119 two interesting cases, illustrated by striking coloured plates which show the marked improvement after treatment, are given—one of cancroid of the nose, the other of a fungating epithelioma of the helix. In both the cure appears to be permanent. An even more striking case is depicted on page 137, showing a huge epithelioma of the parotid region before and after treatment. Other interesting cures are recorded on pp. 156 (leucoplakia), 165 (presternal keloid), 175 (reduction of an unsightly cervical scar the result of old glandular suppuration), 198 (a fluctuating vascular tumour on the neck and face of a young child), 210 (angiomata of the face, also in a child), 226 (unsightly pigmentary nævi), and 218 disfiguring facial nævi). These are only a few of the many striking illustrations that the book contains, two of which we reproduce by courtesy of the publishers, and each case is fully described and a frank *résumé* of the results of treatment given. The indications for treatment are enumerated, and it is specially to be noted that the authors, though rightly enthusiastic about the usefulness of radium therapy in selected cases, are definite in their warnings against subjecting other and unsuitable lesions to the treatment. For instance, in cancer cases, while they report most favourably with regard to epitheliomata, they are pessimistic with reference to cases of oral carcinoma. The non-success in these latter cases

No. 2.—The same Two Months after Treatment with Radium.

they think is to be ascribed to the technical difficulties in treatment in such situations as the mouth. Dr. Wickham's final warning deserves to be widely published. "We desire," he remarks, "definitely to dissociate ourselves from the exaggerated statements on this subject (the treatment of cancer by radium) made by some workers, and although we have gradually become aware of the value of radium in therapeutics, we have always taught that it is of the utmost importance, for the sake of the patients, to proceed with caution and discretion.

The general impression left after reading this able and exhaustive summary of results is that radium is an excellent adjunct in certain cases; that in some it can be held to be a positive cure; but that in no ways can it be described as an infallible panacea for new growths of a malignant nature. The book is one which at the present time should find a wide reading public among members of the profession. Dr. Dore, as translator, has done his work admirably; the publishers, again, have done their share with commendable excellence; the fine type and "get up" of the book make it a pleasure to read. It is, therefore, a book which will do credit to any practitioner's library, and those who are specially interested in the subject with which it deals will find it indispensable. It is *the* text-book on radium.

## Figures and Tables

**No. 1. f1:**
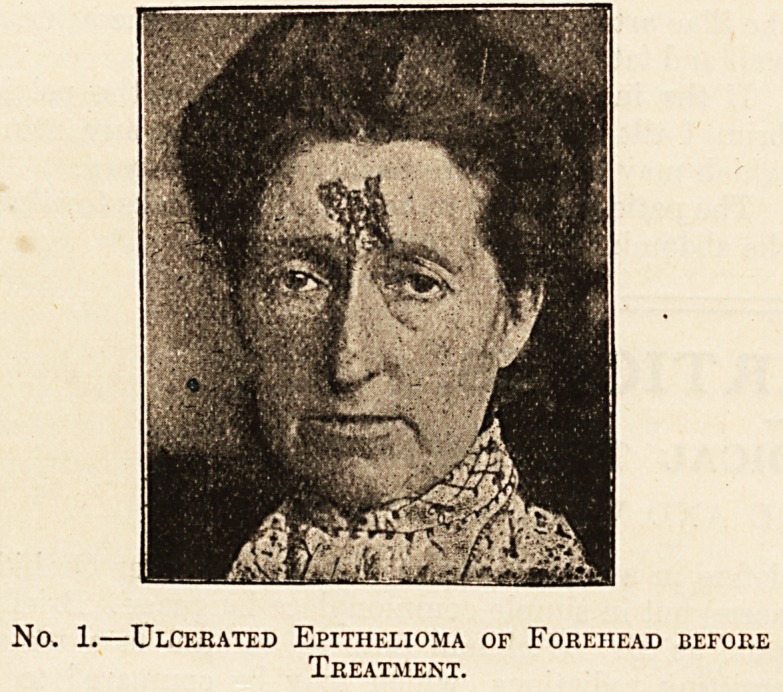


**No. 2. f2:**